# Wireless body area sensor networks based human activity recognition using deep learning

**DOI:** 10.1038/s41598-024-53069-1

**Published:** 2024-02-01

**Authors:** Ehab El-Adawi, Ehab Essa, Mohamed Handosa, Samir Elmougy

**Affiliations:** Department of Computer Science, Faculty of Computers and Information, Mansoura University, Mansoura, 35516 Egypt

**Keywords:** Computer science, Public health

## Abstract

In the healthcare sector, the health status and biological, and physical activity of the patient are monitored among different sensors that collect the required information about these activities using Wireless body area network (WBAN) architecture. Sensor-based human activity recognition (HAR), which offers remarkable qualities of ease and privacy, has drawn increasing attention from researchers with the growth of the Internet of Things (IoT) and wearable technology. Deep learning has the ability to extract high-dimensional information automatically, making end-to-end learning. The most significant obstacles to computer vision, particularly convolutional neural networks (CNNs), are the effect of the environment background, camera shielding, and other variables. This paper aims to propose and develop a new HAR system in WBAN dependence on the Gramian angular field (GAF) and DenseNet. Once the necessary signals are obtained, the input signals undergo pre-processing through artifact removal and median filtering. In the initial stage, the time series data captured by the sensors undergoes a conversion process, transforming it into 2-dimensional images by using the GAF algorithm. Then, DenseNet automatically makes the processes and integrates the data collected from diverse sensors. The experiment results show that the proposed method achieves the best outcomes in which it achieves 97.83% accuracy, 97.83% F-measure, and 97.64 Matthews correlation coefficient (MCC).

## Introduction

Wireless body area network (WBAN) is employed as the fundamental network architecture for various sensor types across diverse applications. These sensors are specifically designed to function on, around, and within the human body. They require very little power and do not need any external assistance. In the healthcare sector, a collection of physical and biological sensors is distributed on the patient’s body to collect information about the patient to be used in monitoring their health status and biological physical activities, checking their physiological measurements or any other objectives, through using WBAN architecture. The sensors distributed on-body used in human activity recognition (HAR) domain^1,2^. A WBAN consists of movable sensors with communication capabilities, managed by a body area networks (BANs) coordinator. Each sensor can be attached to the body^3^. Healthcare devices have undergone a transformation with advancements in microelectronic technology, enabling them to be less intrusive and more wearable or implantable. The fifth-generation communication system offers greater benefits to users, including higher capacity. However, in the absence of BANs, actuator and sensor functions were isolated, leading to inefficient use of communication resources.

**Figure 1:**
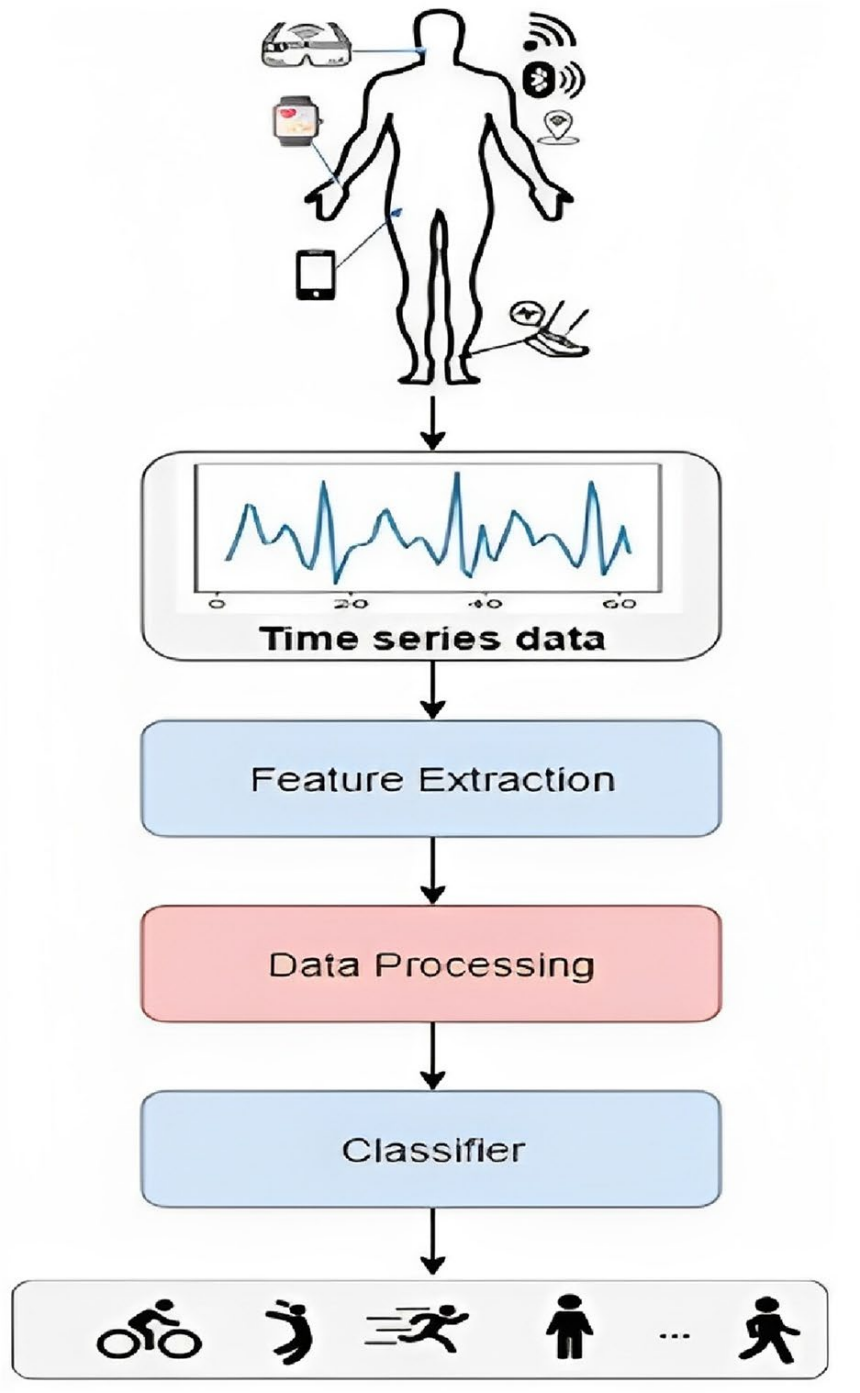
HAR Framework adapted from^10^.

BANs offer numerous advantages, such as providing a solid basis for physical exercise, recuperation, and health tracking^4^. Consequently, it is important to model HAR system in WBAN architecture to achieve high recognition accuracy. In recent years, wearable sensor-based HAR has gained popularity due to the widespread use of mobile devices^5^. Identifying different human activities using sensor data is known as HAR. Due to the Internet of Things (IoT), artificial intelligence (AI), and the rapid development of 6th Generation (6G) mobile networks, HAR is becoming more and more significant in our daily. Particularly in daily activity analysis^6^, video monitoring^7^, identification of gestures^8^, and analysis of gait^9^. While sensor-based activity recognition (AR) is used to evaluate and process data from sensors like accelerometers and gyroscopes, video-based AR primarily processes the video and pictures collected by cameras. Due to its advantages of superior privacy and simplicity, sensor-based AR has become attractive to many researchers’ primary focus. Figure 1 illustrates the HAR framework which consists of four phases: gathering data, preprocessing, and segmentation, extracting features, and classifying activities.

Many recognition models employed in the HAR system are dependence on traditional machine learning (ML) methods, while others are based on deep learning (DL) algorithms. Among the most popular traditional ML algorithms are the decision tree (DT)^11^, random forests (RF)^12,13^, and support vector machine (SVM). The classic ML method has undergone a revolution in the last ten years developed by DL, which has increased performance in various areas such as speech recognition, object identification, image recognition, and natural language processing. DL has significantly improved the performance and reliability of HAR, accelerating its acceptance and applicability in wearable sensor-based applications. Unlike traditional ML techniques that often require manual feature extraction or engineering, a process that demands domain expertise and extensive human effort, DL methods can automatically learn robust features from raw data for specific applications.

Utilizing DL, methods can make the HAR process simpler. The architecture of DL algorithms is composed of stacked layers of neurons that derive hierarchical representations. For each layer, a nonlinear function is applied to generate new feature maps based on the input feature maps from the previous layer. This hierarchical representation enables DL algorithms to autonomously identify the most relevant features specific to the application domain. By minimizing a specific loss function, the DL architecture identifies features and classification boundaries. DL-based methods can learn the features autonomously, eliminating the need for manual feature engineering. DL algorithms such as as convolutional neural network (CNN), recurrent neural networks (RNN)s, Deep Belief Networks, and autoencoders are often used for HAR^8^.

Deep neural networks (DNN) demonstrates the ability to learn meaningful features from raw inputs, even with limited domain expertise. Additionally, when equipped with a substantial network and an ample number of observations, DNNs have been shown to be universal function approximators, capable of approximating almost any function^14^. This high expressive capacity has resulted in a significant surge in HAR-based applications for DL. However, despite the numerous benefits offered by DL, it also has inherent issues and drawbacks-for instance, the vanishing gradient problem. Furthermore, many recognition techniques based on CNN employ one-dimensional convolution kernels^15,16^, which poses challenges in effectively leveraging the rich high-dimensional data characteristics.

Transfer learning is a technique in DL where a pre-trained model on a large dataset is used as a starting point for a new task. One such pre-trained model is the densely connected convolutional networks (DenseNet)^17^, which is a DNN architecture known for its high accuracy and efficiency in computer vision tasks. Transfer DenseNet refers to the application of transfer learning using a pre-trained DenseNet model. The idea is to take advantage of the learned features and parameters of the pre-trained model and adapt them to a new task by fine-tuning the network on a smaller dataset specific to the new task. By using transfer learning with DenseNet, it is possible to achieve higher accuracy with less training time and fewer training data than training a new model from scratch. This is especially useful in scenarios where there is limited labeled data available or when training a new model from scratch is computationally expensive.

There are numerous techniques employed to transform 1-dimensional sensor data into 2-dimensional data through matrix rearrangement. One of these methods involves a straightforward approach of listing and superimposing the data, but it may lose interpretability^18,19^. In^20–22^, the Fourier transform was used to turn the 1-dimensional time series into 2-dimensional time-frequency pictures, which significantly increased the quantity of calculations. In^18^, the Gramian angular field (GAF) transform was used to transform data into a two-dimensional time-frequency image. Sensor-based activity detection systems would inevitably use less computer power and perform calculations more quickly due to wearable sensor devices’ mobility and real-time capabilities.

In this paper, we propose a new HAR system in WBAN based on GAF and DenseNet to convert time series data from one-dimensional into a two-dimensional image and then classify the human activities, that are collected by sensors distributed on the body of the patient, by using DenseNet. The DenseNet offers various appealing benefits, including eliminating the vanishing-gradient issue, enhancing the feature propagation, promoting feature reuse, and much fewer parameters. The GAF method establishes the groundwork for the success of feature extraction while improving the interpretability of the transformation from one-dimensional time series to a 2-dimensional image. The experimental results demonstrate that the proposed HAR approach achieves good performance by combining the GAF algorithm’s features with the structure and benefits of DenseNet, which may significantly increase the multiscale feature extraction capability and the accuracy of activity detection. The contributions of this paper include a AR hybrid approach in WBAN architecture is proposed and developed based on integrating GAF algorithm and DenseNet based on the mobile health (MHEALTH) dataset.

The subsequent sections of this paper are structured as follows: “Literature review” presents related works. “Methods” presents and discusses the proposed methodology. “Results” delves into implementation details. Lastly, “Conclusions and future works” presents and discusses the results obtained.

## Literature review

Batool et al.^23^ proposed an innovative method that employs fused sensors and presents a modified K-Ary entropy classifier algorithm to tackle intricate challenges related to feature selection and classification using RGB-D data. The algorithm is designed to enhance the spacing between intra-substructure nodes within a tree, thereby decreasing the probability of misclassifying the minority class. The proposed model undergoes testing on three benchmark datasets, revealing promising outcomes with performance metrics of 95.05%, 95.56%, and 95.08% for the SYSU-ACTION, PRECIS HAR, and Northwestern-UCLA (N-UCLA) datasets, respectively. K. Abhishek et al.^24^ proposed approach entails deploying a CNN model for the examination of videos recorded by surveillance cameras, with the objective of categorizing the existence of humans in the individual frames of the video. The proposed model undergoes testing on three benchmark datasets, revealing promising outcomes with performance metrics of 92.15%, and 92.83% for Sport Videos in the wild, and UT-interaction datasets, respectively. Boga et al.^25^ proposed a method for HAR using WBAN and DL. The proposed method involves a multi-objective feature selection approach to select the most relevant features from the sensor data collected by the WBAN. These features are then input to a DL model for AR to recognize human activity (HA). Mishra et al.^26^ A system was introduced to facilitate on-device intelligence for Human HAR by utilizing energy harvesting wireless sensor networks (EWSNs). The proposed system utilizes ML algorithms to perform AR on the sensor data collected by the EWSNs. To enable on-device intelligence, the authors propose a hardware platform that integrates the EWSNs with a microcontroller unit and an energy harvesting module. the authors propose present experimental results to demonstrate the effectiveness of the proposed system in accurately recognizing HAs while achieving energy efficiency through the use of energy harvesting. Reich et al.^27^ provide an evaluation of the performance of Bluetooth in a WBAN for practical applications. the authors propose the feasibility of using Bluetooth for transmitting physiological data in a WBAN by evaluating its performance in terms of data rate, power consumption, and latency. the authors propose present experimental results to demonstrate the suitability of Bluetooth for practical WBAN applications, including remote patient monitoring and health tracking. Additionally, the authors discuss the potential challenges and limitations of using Bluetooth in a WBAN, such as interference and security concerns, and proposes possible solutions to address these issues. Fan et al.^28^ proposed a DNN approach for team training using body-worn inertial sensors for HAR. The system aims to provide real-time feedback to team members during training sessions to improve their performance by identifying and analyzing their movements. The proposed method uses a multi-layer neural network (NN) to recognize different activities performed by team members and provides personalized feedback based on their individual performance. they suggest that the system can be used in various team training scenarios, such as sports, military training, and emergency response, to enhance teamwork and overall performance. He et al.^29^ proposed wearable WBAN for HAR. consists of a set of body-worn sensors to detect and measure various physical activities performed by an individual. The gathered data is wirelessly transmitted to a central processing unit, where it undergoes analysis using ML algorithms to discern various activities. The authors propose that the wearable wireless body area network system can be used in various applications, such as healthcare, sports, and elderly care, to monitor and improve human physical activity and overall well-being.

Huan et al.^30^ proposed approach involves the development of a hybrid model that combines CNN and bidirectional long short-term memory (BLSTM) architectures. This model is specifically designed to leverage the PAMAP2 dataset for accurate recognition of HA, this model achieves F1 92.23%. Damirchi et al.^31^ proposed an ARC-Net net based on PAMAP2, and the RealWorld datasets, to predict the activity performed by the subject. It ranges from 89.81% to 90.51% for the PAMAP2 dataset and 90.51% for the RealWorld dataset. Hongji et al.^18^ proposed a new model based on WISDM, UCI HAR, and OPPORTUNITY datasets to improve the HAR method by transforming time-series data into 2D images. By using GAF, they pass the output to the CNN model. The model achieves 95.79%, 89.63%, and 96.04% for the F1 score. and 96.83%, 89.48%, 97.27% for accuracy respectively.

Ahmed et al.^32^ proposed a HAR system that calculates the best wearable sensor data based on MOTIONSENSE, MHEALTH, and the proposed self-annotated IM-AccGyro human-machine datasets, by applying a notch filter to 1D signals and looking at the lower/upper cutoff frequencies. Subsequently, it calculates a variety of composite features, which encompass statistical characteristics, Mel frequency cepstral coefficients, and Gaussian Mixture Model features. The model achieves 88.25%, 93.95%, and 96.83%. Nidhi et al.^10^ proposed a novel approach called “ICGNet” based on MHEALTH and PAMAP2 datasets that makes use of the advantages of CNN and gated recurrent unit (GRU) and can therefore detect local characteristics and long-term relationships in multivariate time series data. The model achieves 99.25% and 97.64%.

Uddin et al.^33^ proposed a solution that leverages CNNs for AR. CNNs are a type of artificial neural network commonly used for analyzing visual data, such as images or video frames. In this paper, the CNN architecture is adapted to process sequential data from body sensors, such as accelerometers or gyroscopes, which provide information about human movements, based on MHEALTH dataset, the model achieves 93.90%. Sheikh et al.^34^ proposed HAR system on USC-HAD, IMSB, and MHEALTH datasets, which collects signal data from inertial sensors, such as gyroscopes and accelerometers used as motion node sensors. The inertial data is first processed using a variety of filters. It derives a multifaced model for statistical, wavelet, and binary features to optimize the occurrence of ideal feature values. Then, in the phase of feature optimization, adaptive moment estimation (Adam) and AdaDelta are added to adopt learning rate patterns. this model achieves 91.25%, 90.91%, and 93.66%. Lingjuan et al.^35^ proposed a model hybrid of CNN and long short-term memory (LSTM) called RG-RP based on MHEALTH and UCI-HAR datasets, which combines the merits of LSTM and CNN. The model achieves 98% and 96.2%. Ha et al.^36^ proposed a CNN-based approach to leverage the spatial and temporal information captured by the accelerometer and gyroscope sensors. By using CNNs, the model can automatically learn relevant features from the raw sensor data without the need for manual feature engineering based on the MEALTH dataset. the model achieved 91.94%. Chen et al.^37^ proposed a model for unbalanced activity detection using a semi-supervised deep model from multimodal wearable sensory data based on MHEALTH, PAMPA2, and UCI-HAR datasets. The researchers proposed a pattern-balanced semi-supervised framework aimed at extracting and preserving diverse latent activity patterns. More specifically, their approach suggests using a pattern-balanced semi-supervised framework for extracting and maintaining different latent activity patterns. The model achieves 94.05%, 83.42%, and 81.32%. Qin et al.^38^ presented HAR architecture to utilize data from multiple sensors with using a hybrid system of GAF and ResNet model based on heterogeneity human activity recognition (HHAR) and MHEALTH datasets. This model provides an accuracy of 93.41% on HHAR dataset and 98.5% on MHEALTH dataset. Table 1 presents a Comparison between the related work for HAR.

**Table 1:** Comparison of different HAR systems.

Ref.	Year	Dataset	Methods/Techniques	Results	
				Accuracy	F1
^36^	2016	MHEALTH	Hybrid between CNN and pff model.	91.94%	–
^35^	2017	USC-HAD	They proposed a recognition model using inertial (gyroscopes and accelerometers) RG-RP	91.25%	–
		MHEALTH		98%	–
		UCI-HAR		96.2%	–
^33^	2018	MHEALTH	CNN model	93.90%	–
^37^	2019	MHEALTH	They carefully chose salient regions human behaviors that are suggestive of using the independence of several sensory from recurrent convolutional attention networks	94.05%	–
		PAMPA2		83.42%	–
		UCI HAR		81.32%	–
^34^	2020	WISDM	They built a new hybrid model (CNN + GAF)	96.83%	95.79%
		IMSB		90.91%	–
		MHEALTH		93.66%	–
^18^	2020	UCI HAR	to improve HAR method and solving the problem of environmental background, camera shielding	89.48%	89.63%
		OPPORTUNITY		97.27%	96.04%
^32^	2020	MOTIONSENSE	Proposed self-annotated IM-AccGyro human-machine using 1D Filter	From 88.25% to 93.95%	–
		MHEALTH		96.83%	–
^31^	2020	PAMAP2	They introduce the ARC-Net framework and suggest using capsules to combine data from various inertial measurement units (IMU) in order to forecast the subject’s activities	From 89.81% to 90.51%	–
		The Real-World		90.51%	–
^38^	2020	HAR	They built a new model to recognition HAR using hybrid model (GAF and ResNet)	93.41%	–
		MHEALTH		98.5%	–
^30^	2021	PAMAP2	They built HAR (using a hybrid model CNN and bidirectional BLSTM)	–	92.23%
		UT-Data		–	98.07%
^10^	2022	MHEALTH	They built HAR (using a hybrid modelCNN and BLSTM)	99.25%	–
		PAMAP2		97.64%	–
		SYSU-ACTION	They create a method using fused sensors and introduce a modified K-Ary entropy classifier algorithm	95.05%	–
^23^	2023	PRECIS HAR		95.56%	–
		Northwestern-UCLA		95.08%	–
^24^	2023	Sport Videos in the Wild	They built CNN model for the examination of videos recorded by surveillance cameras	92.15%	–
		UT-interaction		92.83%	–

## Methods

### Dataset description

**Figure 2:**
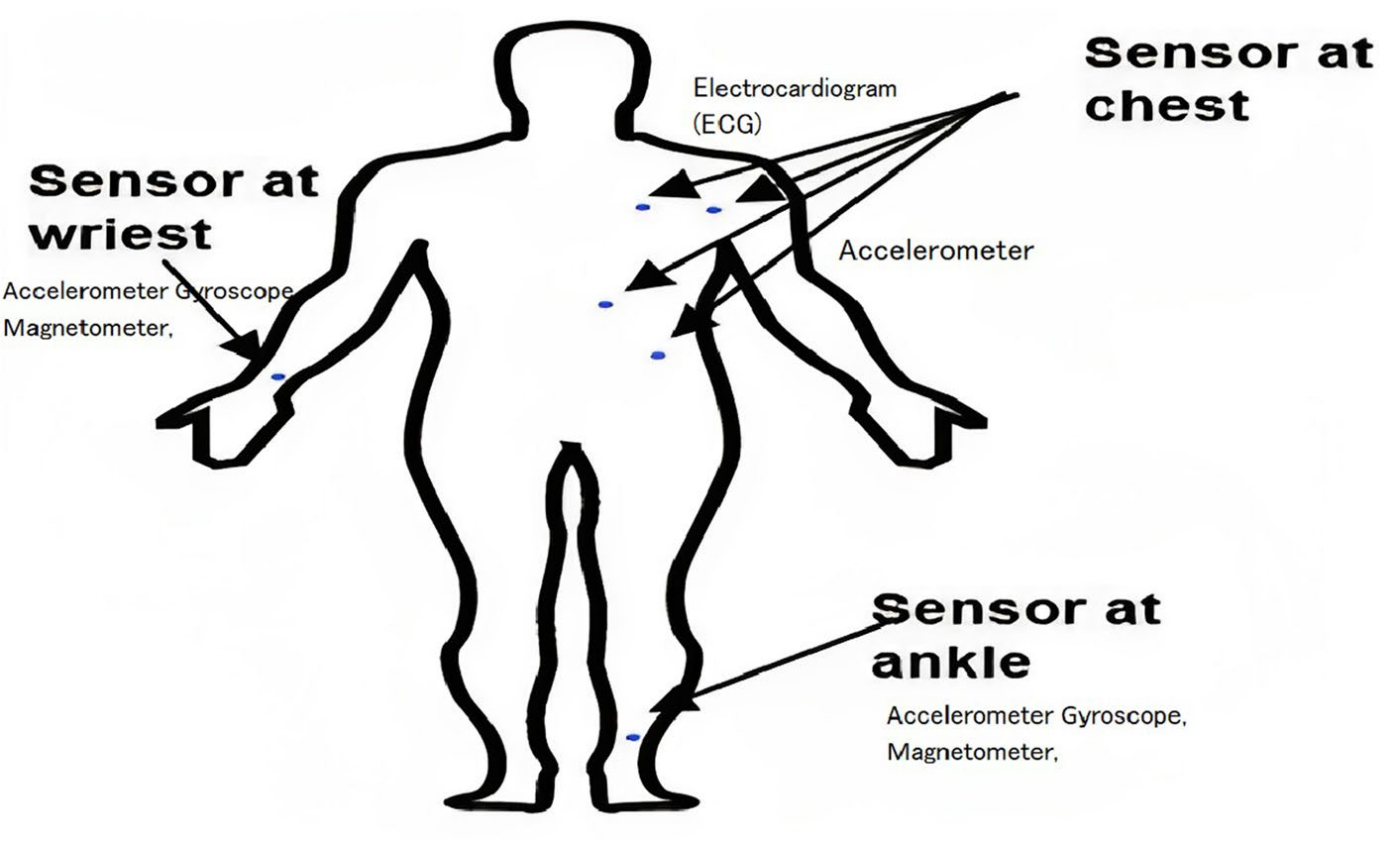
The position of the sensor to collect data for the MHEALTH dataset^40^.

The UCI repository makes the MHEALTH dataset^39^ available; it contains information on 12 activities carried out by ten people. This dataset includes the following activities: stair climbing, cycling, frontal arm elevation, jogging, back and front jumps, knee bending (crouching), lying down, running, sitting and relaxing, standing still, forward-bent waist, and walking. The data was collected by using sensors positioned at the body parts: chest, right wrist, and left ankle; Fig. 2 depicts where the locations of these sensors.

In this work, we use the gyroscope, accelerometer, and magnetometer sensors. The characteristics that are recorded by the accelerometer, gyroscope, magnetometer, and electrocardiogram (ECG) are (*axe*, *ay*, *az*), (*gx*, *gy*, *gz*), and (*mx*, *my*, *mz*), respectively, in all three x, y, and z-direction. The combination of several sensors makes it possible to measure the motion felt by various body parts, like the rate of rotation, acceleration, and magnetic field direction. ECG readings can track your essential health, the effects of certain activities, and more. Additionally, recordings of ECG signals were made. Lead1 and lead2 signals on the ECG have two characteristics. Accelerometer and ECG signals were captured at the chest, and accelerometer, gyroscope, and magnetometer signals were recorded at the right wrist and left ankle. Thus, for all three places’ qualities and features, a total of 23 were recorded. A sampling rate of 50 Hz was used to record all sensing modalities. Then the collected sensor data is transmitted from the individual sensors to a central unit. This central unit could be the user’s smartphone, a dedicated wearable device, or another computing device capable of processing and transmitting data.

Sensors in the MHEALTH dataset typically use wireless communication protocols to transmit data. Common protocols include Bluetooth, Wi-Fi, or other short-range communication technologies. These protocols allow for efficient and wireless data transfer between the sensors and the central unit.

### The proposed methodology

**Figure 3:**
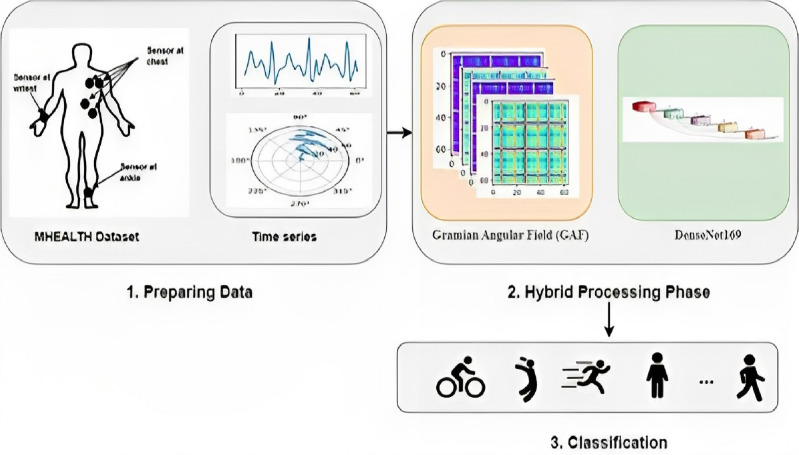
The proposed hybrid HAR system in WBAN architecture based on GAF algorithm and DenseNet169 model.

This paper proposes a new HAR hybrid system in WBAN architecture based on GAF algorithm and DenseNet169 model, in which Fig. 3, shows the main steps of this architecture are as follows:

### Preprocessing data

Most of the sensor’s initial data set consists of 1-dimensional time series^18^

In the application of 2-dimensional, it is typically necessary to transform 1-dimensional time series into a format resembling 2-dimensional images. In the first step, the mobile health dataset is stored and represented as a CSV file and performs the following steps:

1. Resampling: The majority class (activity label = 0) in the dataset is downsampled to have 30,000 samples using the resample function from sklearn. utils. This is done to balance the class distribution in the dataset.
2. Outlier removal: the features in the dataset that have data points outside the 98% confidence interval are dropped using a for loop that iterates over each column except the last two columns (Activity and subject).
3. Train-test split: The data is divided into train and test sets based on the subject column. Data corresponding to subjects 9 and 10 are considered as the test set, while the remaining data is considered as the train set.
4. Time series dataset creation: A function named a window, which has a fixed size or a sliding nature, depending on the specific application or task. A fixed-size window selects a predetermined number of consecutive data points, while a sliding window moves across the dataset in steps, selecting a new subset of data for each step

Overall, the data is prepared dataset for time series modeling by balancing class distribution, removing outliers, and creating time series datasets for sequence modeling.

### Gramian angular algorithm (GAF)

The bearing vibration signal is periodic in rotating machinery. It is challenging to directly extract the bearing defect features from the time-domain signal because random noise affects the periodic vibration signal. With GAF. Time-domain signals can be separated into characteristic and interference signals while maintaining their temporal link. In terms of human behavior recognition (HAR) and ECG signal monitoring, GAF has made some progress recently^41^. Consider a one-dimensional time series $X=\{x_1,x_2,\dots , x_N\}$ of *N* observations. According to the GAF algorithm, the observation $x_i; i=1,\dots , N$ are normalized to range $[-1,1]$ or [0, 1] such that the normalized observations are given by^18^:

1 $$\begin{aligned} {\hat{x}}_i^{[-1,1]}=\frac{(x_i-max(X))+(x_i-min(X))}{max(X)-min(X)} \end{aligned}$$

or

2 $$\begin{aligned} {\hat{x}}_i^{[0,1]}=\frac{(x_i-max(X))+(x_i-min(X))}{max(X)-min(X)}, \end{aligned}$$

respectively. Thus, a normalized one-dimensional time series is obtained, denoted by ${\hat{X}}$. Next, ${\hat{X}}$ is converted to the polar coordinate system by taking the inverse cosine of each normalized observation ${\hat{x}}_i; i=1,\dots ,N$ to be the angle and *i*/*N* to be the radius. The observations in the polar coordinate system are given by^18^:

3 $$\begin{aligned} {\left\{ \begin{array}{ll} \theta _i=\arccos {{\hat{x}}_i},\\ &{} i=1,\dots , N.\\ r_i=\frac{i}{N}, \end{array}\right. } \end{aligned}$$

Note that, when normalizing the data to the range $[-1,1]$ the rang of $\theta _i$ is $[0,\pi ]$, whereas the range of $\theta _i$ related to the data range [0, 1] is $[0,\pi /2]$.

Finally, Gramian angular summation field (GASF) and Gramian angular difference field (GADF) are obtained to determine the time correlation between the sampling points from the angle perspective. GAF algorithm is applied for converting the one-dimensional time series into a two-dimensional image. Applying computer vision technology to the study of time involves scaling, coordinate axis transformation, and trigonometric function stages. The time series is converted into a polar coordinate system using (3). Using (4) and (5), you may get the GADF and GASF images, respectively. The GASF and GADF are, respectively, given by^18^

4 $$\begin{aligned} \text {GASF} = \begin{bmatrix} \cos {(\theta _1+\theta _1)}&{}\cdots &{} \cos {(\theta _1+\theta _N)}\\ \cos {(\theta _2+\theta _1)}&{}\cdots &{} \cos {(\theta _2+\theta _N)}\\ \vdots &{}\ddots &{}\vdots \\ \cos {(\theta _N+\theta _1)}&{}\cdots &{} \cos {(\theta _N+\theta _N)} \end{bmatrix}, \end{aligned}$$

and

5 $$\begin{aligned} \text {GADF}= \begin{bmatrix} \sin {(\theta _1-\theta _1)}&{}\cdots &{} \sin {(\theta _1-\theta _N)}\\ \sin {(\theta _2-\theta _1)}&{}\cdots &{} \sin {(\theta _2-\theta _N)}\\ \vdots &{}\ddots &{}\vdots \\ \sin {(\theta _N-\theta _1)}&{}\cdots &{} \sin {(\theta _N-\theta _N)} \end{bmatrix}. \end{aligned}$$

### DenseNet

DenseNet is a CNN architecture proposed Huang et al.^17^ in 2017. It is designed to address the vanishing gradient problem that can occur in very DNNs by densely connecting all layers.

Every layer in DenseNet receives the feature maps of all the preceding layers and passes its own feature maps to all subsequent layers. This creates a dense connectivity pattern between layers, with information flowing through many paths, which helps to reduce the risk of information loss due to vanishing gradients. DenseNet models consist of dense blocks, which are composed of multiple convolutional layers with batch normalization and ReLU activation, followed by a concatenation operation that combines the feature maps from all preceding layers. These dense blocks are connected by transition layers, which include a pooling layer to reduce the spatial dimensions of the feature maps and a convolutional layer to reduce the number of channels. DenseNet models have achieved state-of-the-art performance on a variety of image classification tasks, object detection, and segmentation. The architecture of the DenseNet model consists of dense blocks and transition layers, as described below:^17^

1. **Input Layer**: The input layer of a DenseNet model takes the image as input.
2. **Convolutional Layer**: The input is passed through a single convolutional layer with a small kernel size (that is 3x3), followed by batch normalization and ReLU activation.
3. **Dense Block**: The output of the first convolutional layer is passed through a dense block, which consists of multiple convolutional layers with the same kernel size and a number of filters. Each layer in the dense block takes as input the concatenated feature maps of all preceding layers in the block. The output of each layer is passed through batch normalization and ReLU activation before being concatenated with the previous layer’s output.
4. **Transition Layer**: After the dense block, a transition layer is added to reduce the number of feature maps by using 1x1 convolution and downsampling the spatial dimensions using average pooling.
5. **Repeat Dense Block and Transition Layer**: The above two steps (dense block and transition layer) are repeated multiple times to create a deep neural network architecture.
6. **Global Average Pooling Layer**: A global average pooling layer is added after the last dense block to reduce the spatial dimensions of the feature maps to a vector of size 1x1xk, where k is the number of filters in the last dense block.
7. **Fully Connected Layer**: Finally, a fully connected layer with softmax activation is added to produce the output probabilities for the different classes.

The dense connectivity pattern between layers in a DenseNet model allows for efficient parameter sharing, which leads to a compact model with fewer parameters compared to other deep neural network architectures. This, in turn, leads to faster training and reduced risk of overfitting^42^. There are several types of DenseNet models that have been proposed, including:

1. **DenseNet-121**: This is the smallest and most widely used DenseNet model, which contains 121 layers. It has about 7 million parameters and is suitable for applications with limited computational resources.
2. **DenseNet-169**: This model contains 169 layers and has about 14 million parameters. It is deeper and more complex than DenseNet-121, which can lead to improved performance on more challenging tasks.
3. **DenseNet-201**: This model contains 201 layers and has about 20 million parameters. It is even deeper and more complex than DenseNet-169, which can provide better accuracy on more complex datasets.
4. **DenseNet-264**: This model is the deepest and most complex DenseNet architecture, with 264 layers and about 33 million parameters. It is designed for very challenging tasks that require a lot of computational power.

The proposed method steps are stated in Algorithm 1.

**Algorithm 1:**
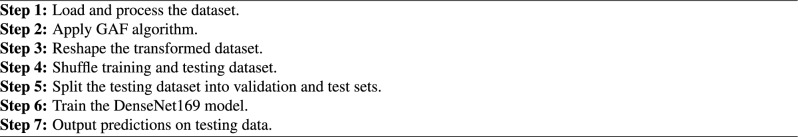
The steps of the proposed method based on a hybrid GAF + DenseNet169.

## Results

### Evaluation measures

To evaluate the proposed work, different measures given in (6)–(7) are used based on constructing a confusion matrix that summarized the following terms: Correct prediction is regarded as true positive (TP), while a prediction that is negative and is made as such is viewed as true negative (TN). False Positive is when something is categorized as negative but false positive (FP). This is regarded as False-Negative if it is positive and classified as false negative (FN). Accuracy is the rightly prognosticated sample rate. It’s the rate between rightly prognosticated samples to the total number of samples due to its straightforward meaning. It is considered the most most habituated criteria in the field of machine literacy evaluation, as illustrated in Eq. (6)^43^

6 $$\begin{aligned} ACC.=\frac{{TP}+{TN}}{{TP}+{TN}+{FP}+{FN}} \end{aligned}$$

Matthews correlation coefficient (MCC) stands for Matthews Correlation Coefficient, used to assess the quality of binary classification models, in which it is computed as presented in Eq. (7).

7 $$\begin{aligned} \text {MCC} = \frac{{\text {TP} \times \text {TN} - \text {FP} \times \text {FN}}}{{\sqrt{{(\text {TP} + \text {FP}) \times (\text {TP} + \text {FN}) \times (\text {TN} + \text {FP}) \times (\text {TN} + \text {FN})}}}} \end{aligned}$$

F1-measure displays the harmonic mean between recall and precision as presented in Eq. (8)^43^.

8 $$\begin{aligned} F1-measure=\frac{2\times Precision\times Recall }{Precision+Recall} \end{aligned}$$

### Enivornment and parameters setting

In this section, the parameters to set up the environment of the proposed architecture, as given in Table 2.

**Table 2:** Environment experimentation.

Environment	Parameters
System	Intel(R) Xeon(R) CPU @ 2.00 GHz CPU MHz : 2199.998. GPU Name: Intel(R) Xeon(R) CPU 2.00 GHz
Tools and Library	Kaggle - Tensorflow - Keras - OpenCV - Pandas Numpy - Skearn.

### Experimentation and results

**Figure 4:**
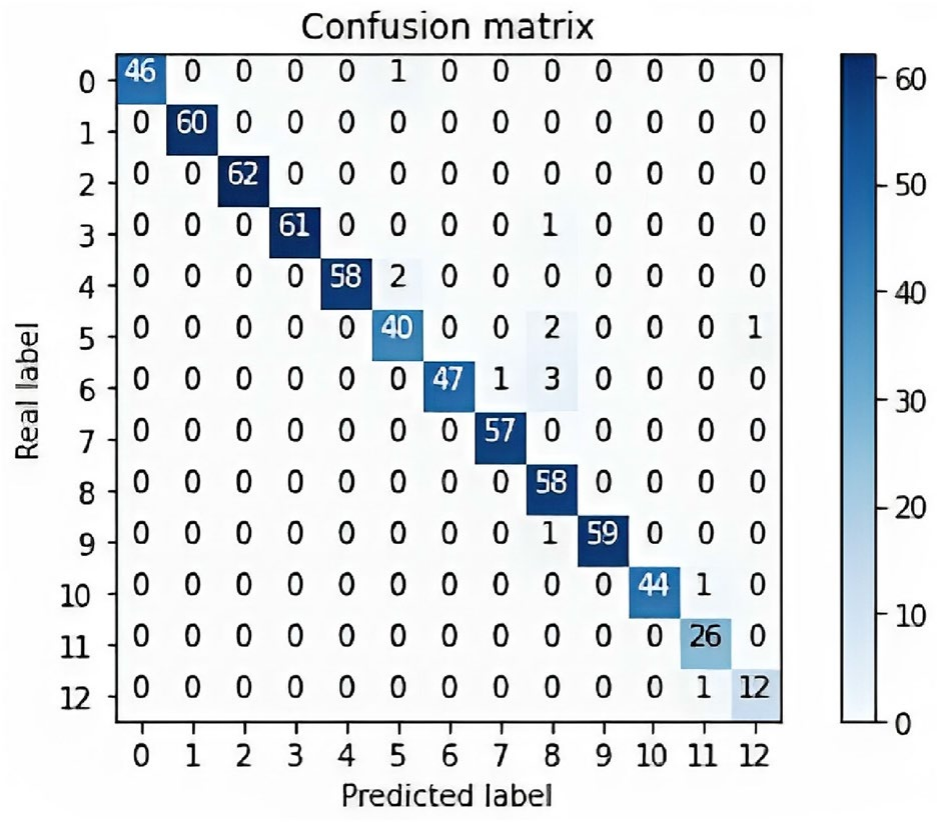
Confusion matrix for the model output.

The model is implemented using Keras and TensorFlow. The input is concatenated with itself three times (300, 300, 3) using the Concatenate layer to facilitate the use of the transfer learning. This concatenated output is then passed as input to the pre-trained DenseNet169 model, which has been initialized with ImageNet weights. A Dense layer with 355 units and a ReLU activation function is added on top of the output of the DenseNet model. A Dropout layer with a rate of 0.2 is then applied to the output of the Dense layer. The output is then flattened using the Flatten layer. Finally, a Dense layer with 13 units with a softmax activation function is added to the flattened output, which outputs the predicted probabilities for each of the 13 classes as shown in Algorithm 1. The entire model is compiled using the Adam optimizer, and categorical cross-entropy loss function. Figure 4 presented the Confusion matrix.

**Figure 5:**
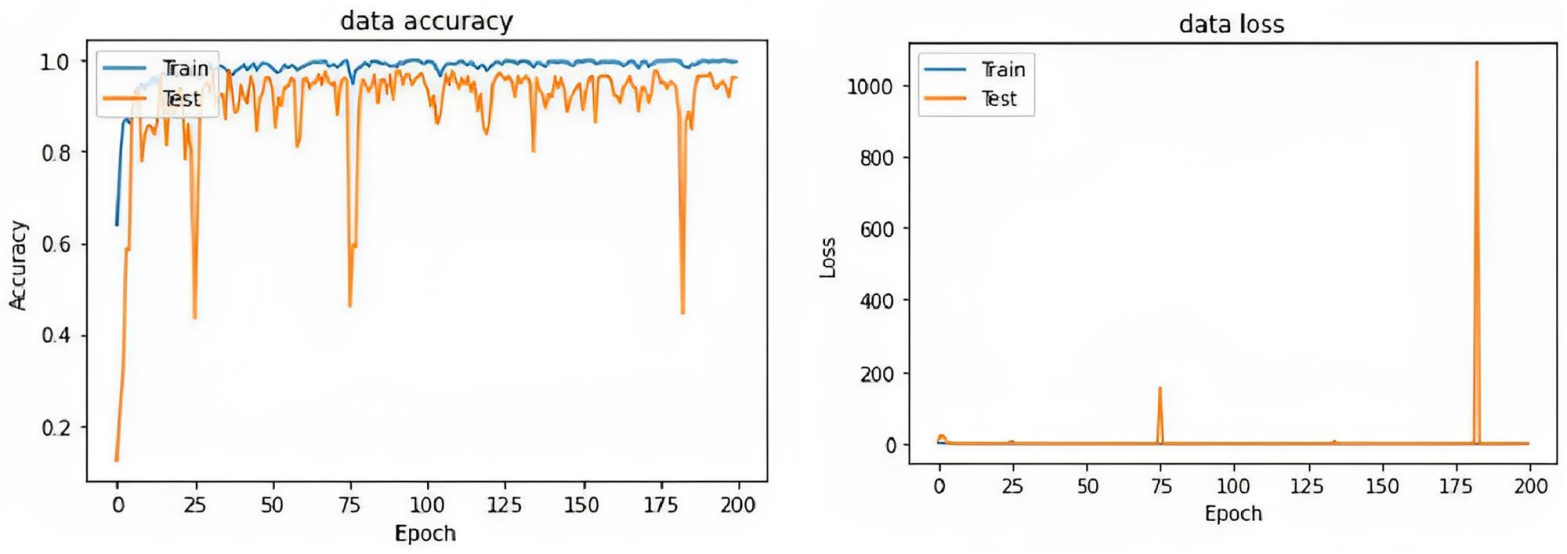
Plots of accuracy curves for the dataset.

DenseNet169 model applied to the MHEALTH dataset achieves an accuracy of 97.83% and an F1 of 97.83% and 97.64% for MCC. Figure 5 shows plots of accuracy and loss data curves. Table 3 shows the performance of a GAF + DenseNet169 model on a classification task across different hyperparameter configurations. The model’s accuracy, F1 score, and MCC are reported for each configuration. The results indicate that the best performance was obtained with an image size of 300, a dense layer of 355, and a batch size of 35, resulting in accuracy, F1 score, and MCC of 97.83%, 97.83%, and 97.64%, respectively. This indicates that larger image sizes and dense layers may improve the model’s performance. The lowest accuracy (86.18%), F1 score (86.18%), and MCC (85.21%) are achieved with an image size of 256, dense layer size of 256, and a batch size of 32. Examining the various configurations and their corresponding performance metrics:

1. When the image size is 300, the models generally perform better, with most configurations achieving high accuracy, F1 score, and MCC. In this image size category, the best configuration has a dense layer size of 355 and a batch size of 35, achieving 97.83% accuracy.
2. For an image size of 128, the performance tends to be lower compared to the 300 image size category, with the highest accuracy and F1 score of 94.41% achieved by the configuration with a dense layer size of 350 and a batch size of 32. This configuration also has an MCC of 93.98.
3. In the image size category of 256, the models have varying performances. The best configuration in this category has a dense layer size of 300 and a batch size of 32, achieving 93.32% accuracy, 93.32% F1 score, and 92.78% MCC.
4. The configurations with a dense layer size of 512 don’t seem to perform as well as those with smaller dense layer sizes. This could be due to overfitting or increased complexity, which might require more training data or a more optimized architecture.
5. The results also indicate that increasing batch size from 32 to 35 can lead to improving performance, as seen in configurations with image size 300 and dense layer sizes of 375 and 350.
6. The configurations with an image size of 300 perform consistently better than those with smaller image sizes, which suggests that the increased resolution might be beneficial for the specific task at hand. However, it would be interesting to test even larger image sizes to evaluate if this trend continues or if there is an optimal size for the best performance.
7. Regarding dense layer size, it seems to be a sweet spot around 350–380 for an image size of 300. For image size 128, the dense layer size of 350 achieves the best performance. However, the performance decreases with a dense layer size of 512 for both image sizes 128 and 256. Depending on the specific task and dataset, this may indicate that the dense layer size should be tuned carefully to avoid overfitting or underfitting.
8. Batch size has less impact on the performance compared to image size and dense layer size. However, in some configurations, increasing the batch size from 32 to 35 has improved the results, suggesting that it might be worth exploring a wider range of batch sizes to find the optimal value for the specific task.

The GAF+DenseNet169 model is a deep learning model that combines the GAF representation with a DenseNet169 architecture for image classification. The GAF representation is a feature extraction method that transforms a time series signal (such as an image) into an image-like representation that can be used in CNNs. This method has been shown to be effective in capturing the underlying patterns and correlations in time series data. DenseNet169 is a popular CNN architecture that uses densely connected layers to improve gradient flow and reduce the number of parameters compared to other CNN architectures such as visual geometry group (VGG) or ResNet. In this work, the GAF+DenseNet169 model was trained and evaluated on a classification task, with the goal of accurately classifying images. The hyperparameters of the model, such as image size, dense layer size, and batch size, were varied to determine their impact on the model’s performance.

**Table 3:** Results for of applying the proposed Hybrid model for GAF and DenseNet169 on MHEALTH dataset using Adam optimizer.

#	ImgSize	DenseLayer	Batchsize	Accuracy	F1	MCC
1	300	380	35	96.58	96.58	–
2	300	375	35	97.2	97.2	–
3	300	350	35	97.36	97.36	–
4	300	355	35	97.83	97.83	97.64
5	300	250	32	96.43	96.43	96.12
6	300	512	32	91.77	91.77	91.24
7	300	360	32	96.27	96.27	–
8	128	256	32	92.55	92.55	91.91
9	128	350	32	94.41	94.41	93.98
10	128	512	32	90.37	90.37	89.66
11	256	256	32	86.18	86.18	85.21
12	256	300	32	93.32	93.32	92.78
13	256	512	32	90.22	90.22	89.46

Table 4 shows the evaluation results of the test cases for each individual sensor alone. The four sensors from the MHEALTH dataset are evaluated to determine the best sensor that contributes more to the proposed method. The information is presented below in a list as follows:

**Table 4:** Evaluation results of the constructed test cases for each sensor.

#	ImageSize	DenseLayer	Sensor 1			Sensor 2			Sensor 3			Sensor 4		
			ACC.	F1	MCC	ACC.	F1	MCC	ACC.	F1	MCC	ACC.	F1	MCC
1	150	380	66.15	66.15	63.52	57.14	57.14	53.64	79.50	79.50	77.91	53.11	53.11	49.43
2	150	360	75.31	75.31	73.23	58.85	58.85	57.62	89.96	86.96	85.86	52.95	52.95	48.86
3	150	375	73.29	73.29	71.10	60.87	60.87	57.62	83.07	83.07	81.69	51.09	51.09	47.50
4	150	512	75.62	75.62	73.71	58.70	58.70	55.26	76.24	76.24	74.31	55.28	55.28	51.81
5	150	512	74.07	74.07	72.08	60.56	60.56	57.14	75.47	75.47	73.88	55.59	55.59	52.51
6	150	360	69.72	69.72	67.17	55.75	55.75	51.98	75.00	75.00	72.98	56.99	56.99	53.61
7	150	380	75.16	75.16	71.48	55.75	55.75	51.99	77.95	77.95	76.29	52.33	52.33	48.88
8	150	375	77.17	77.17	75.35	55.75	55.75	51.99	77.33	77.33	75.60	57.14	57.14	53.66
9	128	512	69.88	69.88	67.66	61.80	61.80	58.56	70.50	70.50	68.44	57.92	57.92	54.49

1. In experiment 8, Sensor 1 was tested using an image size of 150, a Dense layer of 375, and a batch size of 32. The results showed an ACC. of 77.17%, an F1 score of 77.17%, and an MCC of 75.35%.
2. For Sensor 2, experiment 3 utilized an image size of 150, a Dense layer of 375, and a batch size of 35. The corresponding metrics were an accuracy of 60.87%, an F1 score of 60.87%, and an MCC of 57.62%.
3. In the case of Sensor 3, experiment 2 used an image size of 150, a Dense layer of 360, and a batch size of 35. The achieved values were an accuracy of 86.96%, an F1 score of 86.96%, and an MCC of 85.86%.
4. Lastly, for Sensor 4, experiment 9 involved an image size of 128, a Dense layer of 512, and a batch size of 32. The recorded metrics were an accuracy of 57.92%, an F1 score of 57.92%, and an MCC of 54.49%.

To summarize, Sensor 3 yields the best results compared to other sensors. However, its individual performance is still far behind the combination of all sensors. This indicates that while Sensor 3 is superior, it is the collective data from all sensors that provide the most optimal outcome. The comparison results of the various models are given below for HAR in which each model is evaluated based on accuracy as shown in Table 5

**Table 5:** Results of comparison of different approaches.

Model no.	Approaches	Accuracy (%)	F1-score (%)
1	Gaussian-Kernel principal components Analysis (PCA)+CNN^33^	93.90	–
2	DT classifier + binary grey wolf optimization (BGWO)^32^	93.95	–
3	Adam + maximum entropy Markov model (MEMM)^34^	90.91	–
4	CNN-pff model^36^	91.94	–
5	Hybrid model between LSTM-CNN^35^	95.56	–
6	Semisupervised deep model^37^	94.05	–
7	GAF+ DenseNet169 (the proposed model)	97.83	97.83

1. Model 1 combines PCA with a CNN for feature extraction and AR.
2. Model 2 combines a decision tree classifier with the BGWO optimization algorithm for AR.
3. Model 3 combines the Adam optimizer with MEMM for AR.
4. Model 4 model in which no details about the specific architecture or method employed in this model are provided.
5. Model 5 combines LSTM and CNN architectures for AR.
6. Model 6 incorporates semisupervised learning techniques to leverage both labeled and unlabeled data for AR.
7. GAF+ DenseNet169 (the proposed model) utilizes GAF in combination with the DenseNet169 architecture, which is deep convolutional neural networks (DCNN), for AR. It’s worth noting that the table provides limited information, and the performance of the models may depend on various factors, such as the specific dataset used, the size of the training set, the preprocessing techniques applied, and other implementation details. Additionally, the proposed model (GAF+DenseNet169) achieves the highest accuracy, according to the table.

This paper introduces a DL network architecture that aims to recognize human activities using mobile sensor data. The proposed approach focuses on encoding time series into GAF images by combining global and local features. This innovative processing technique allows the training of the model using popular residual networks for image recognition. The obtained results, based on MHEALTH dataset, show that the proposed gives better accuracy and F1-measure than the other compared work.

## Conclusions and future works

Nowadays, there is a growing interest in sensor-based HAR has been propelled by the widespread adoption of IoT and wearable technologies. These technological trends not only offer unparalleled convenience in our daily lives but also address concerns related to privacy, making them increasingly integral to modern living. In parallel, the advent of DL algorithms has ushered in a new era of possibilities, particularly in the context of HAR. The inherent capacity of DL algorithms to autonomously extract high-dimensional information has proven to be transformative, enabling end-to-end learning and enhancing the accuracy of AR systems. However, to build robust computer vision, especially in the realm of CNNs, encounters significant challenges. Factors such as external background interference, camera shielding, and other environmental variables can impede the efficacy of these vision-based systems. This highlights a critical gap where sensor-based HAR, with its intrinsic ability to mitigate such challenges, emerges as a compelling alternative.

Our proposed approach seeks to address these challenges through the integration of two DL models tailored specifically for sensor-based HAR. At the core of our methodology is the utilization of the GAF algorithm, a powerful tool for transforming 1-dimensional time series data obtained from sensors into 2-dimensional images. This transformation is pivotal in capturing nuanced patterns and temporal dependencies in the data, providing a comprehensive representation for subsequent analysis. Subsequently, we deploy the DenseNet model, known for its depth and interconnectedness, to perform accurate classification of various human activities. The unique architecture of DenseNet facilitates the automated processing of information acquired from diverse sensors. This integration not only enhances the efficiency of the recognition process but also contributes to the adaptability of the system to different sensor modalities.

In our experimental results, we conducted tests on the MHEALTH public activity dataset, a well-established benchmark for HAR research. The evaluation metrics employed, including accuracy, recall, and F-measure, serve as robust indicators of the effectiveness of our proposed method. The attained results underscore the prowess of our approach, with an impressive accuracy rate of 97.83%, an F-measure of 97.83%, and a MCC of 97.64%.

In future work, we will explore the potential of another DL model augmented with a feature selection optimizer. This strategic enhancement aims to further refine results by selecting and prioritizing relevant features, thereby improving both predictive performance and interpretability. Additionally, this optimization effort seeks to streamline computational complexity, reducing time requirements and minimizing loss in the HAR system. Overall, these endeavors contribute to the ongoing evolution of sensor-based HAR systems, enhancing their reliability and applicability in real-world scenarios.

## Data Availability

The dataset used during the current study is available in the UCI Machine Learning Repository, MHEALTH Dataset Link.
